# Treatment of an aggressive aneurysmal bone cyst with percutaneous injection of polidocanol: a case report

**DOI:** 10.1186/1752-1947-8-450

**Published:** 2014-12-20

**Authors:** Otte Brosjö, Panagiotis Tsagozis

**Affiliations:** 1 Section of Orthopaedics, Department of Molecular Medicine and Surgery, Karolinska Institute, Stockholm, S-17176 Sweden; 2 Department of Orthopaedics, Karolinska University Hospital, Stockholm, S-17176 Sweden

**Keywords:** Aggressive aneurysmal bone cyst, Polidocanol, Sclerotherapy

## Abstract

**Introduction:**

Aneurysmal bone cysts are benign tumours that usually present in childhood. Aggressive forms have been described, which are often treated with surgery that entails major resection and reconstruction. Polidocanol sclerotherapy has recently been reported to have excellent results and promises to replace operative treatments, but its efficacy in the case of aggressive aneurysmal bone cysts has not been documented.

**Case presentation:**

An 18-year-old woman from Sweden presented with pain in her shoulder and a rapidly progressing cystic bone lesion. The differential diagnosis was a rare, aggressive form of aneurysmal bone cyst or a sarcoma of the proximal humerus. She was successfully treated using sequential percutaneous injections of polidocanol after exclusion of malignancy.

**Conclusions:**

Management of aggressive aneurysmal bone cysts has thus far relied on open surgery. We propose that non-operative treatment with polidocanol is efficient even in the aggressive form of the aneurysmal bone cyst.

## Introduction

Aneurysmal bone cysts (ABCs) demonstrate a spectrum of clinical presentations, from the latent to the active form and finally the rare aggressive variant [1]. The latter is sometimes extremely difficult to differentiate from telangiectatic osteosarcoma. A variety of treatments is available for ABCs, ranging from curettage (with or without filling of the cavity with polymethylmethacrylate) or autologous *en block* excisions with reconstruction of the skeletal defect with autograft or allograft, cryosurgery, selective embolization of the feeding arteries or radiation [1–4]. Most authors have relied on a more radical resection and subsequent reconstruction of the skeletal defect in order to treat aggressive ABCs [5–8]. Healing has also been achieved by selective arterial embolization [9] or radiotherapy, combined or not with cryosurgery [1, 10].

Polidocanol sclerotherapy is a safe and effective treatment method [11, 12] that has gained popularity among orthopaedic surgeons, but its applicability in the management of aggressive ABCs has not been reported. We report for the first time in the medical literature the successful non-operative treatment of an aggressive ABC using polidocanol sclerotherapy.

## Case presentation

**Figure 1:**
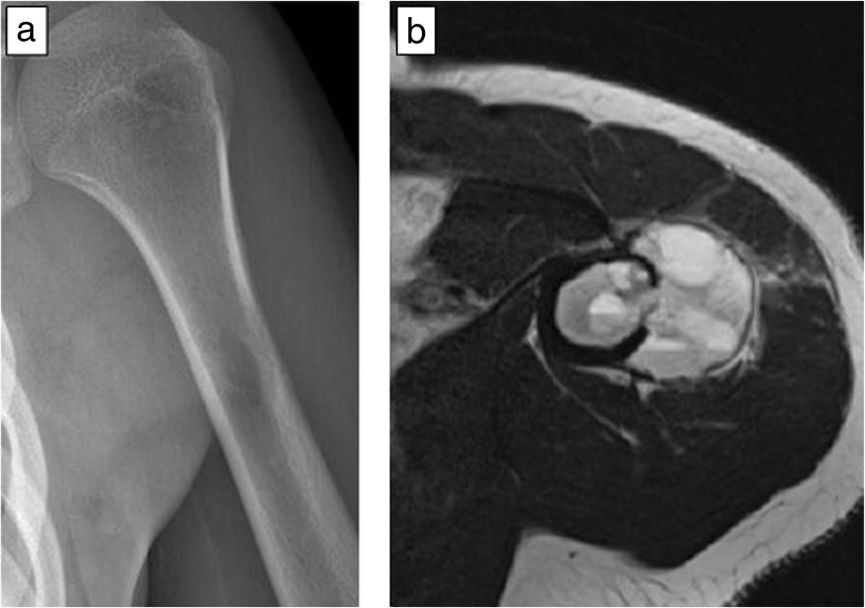
**Plain radiograph of the patient showed an osteolytic lesion in the proximal humeral diaphysis (a), whereas magnetic resonance imaging revealed an expansile tumour that occupied the medullary cavity and gave rise to a soft tissue component (b).**

**Figure 2:**
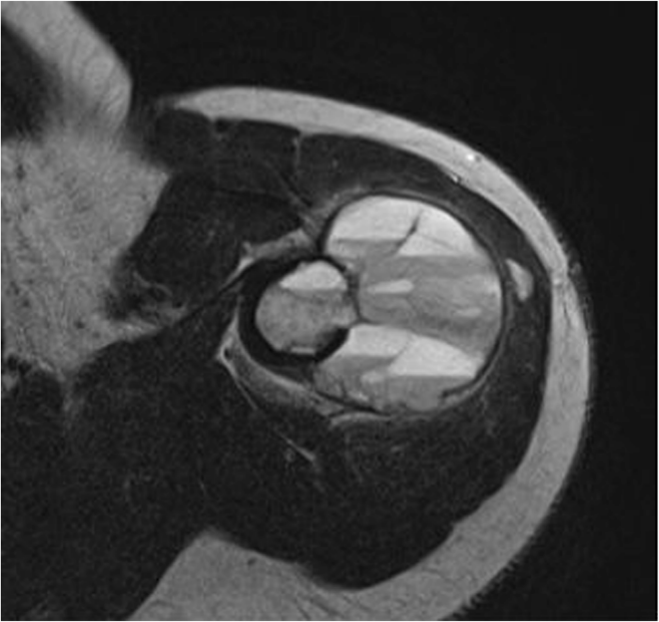
**Repeat magnetic resonance imaging 6 weeks after the initial one, showing considerable growth of both the intraosseous as well as the soft tissue component of the aneurysmal bone cyst (approximately 30% and 300% respectively).**

**Figure 3:**
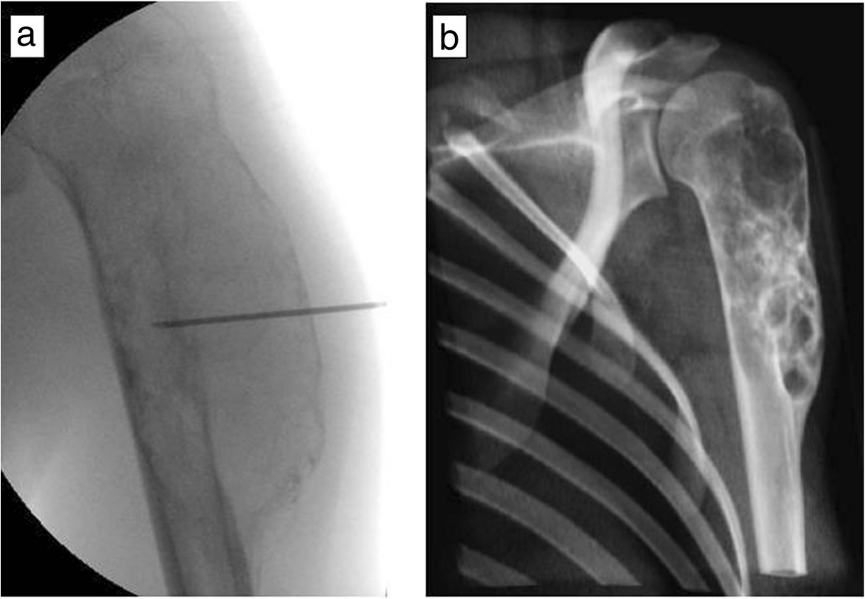
**Fluoroscopy picture taken during the 5th injection of polidocanol, showing proper placement of the needle and signs of sclerosis (a), and graphical reconstruction of a computed tomography scan of the proximal humerus, 18 months after the last treatment, showing consolidation of the lesion (b).**

An 18-year-old woman from Sweden was admitted to our institution due to a painful mass in her left shoulder, discovered 2 months ago, and impairment in range-of-motion (ROM). An ultrasound examination by the admitting primary care physician revealed a vascularized tumour. During physical examination, a palpable mass was present in close proximity to her deltoid muscle. A plain X-ray examination of her left humerus showed an osteolytic lesion (Figure 1a) and magnetic resonance imaging (MRI) revealed a 5.5cm tumour which had a discrete soft tissue component (Figure 1b). Fine needle aspiration biopsy was inconclusive, most suggestive of myositis ossificans. Overall, telangiectatic osteosarcoma could not be excluded, a fact that led us to an open biopsy. The pathology report verified the diagnosis of ABC, without any evidence of malignancy. A repeat MRI, approximately 6 weeks after the first scan, however, showed a clear progression of both the intraosseous and the extraosseous components of the tumour, raising again the suspicion of sarcoma (Figure 2). This led to a second open biopsy, and the second pathology report was also consistent with an ABC. The diagnosis of an unusually aggressive ABC was set, and polidocanol sclerotherapy was initiated. She received six consecutive injections of 240mg of polidocanol under general anaesthesia and fluoroscopic guidance at approximately 3-week time intervals (Figure 3a), until symptoms subsided and convincing radiological findings of healing of the lesion were observed. She had no symptoms at follow-up 3 months after the last injection, and her shoulder ROM was normalized. She remains symptom-free 1.5 years after the last injection, and there is radiologic consolidation of the lesion (Figure 3b).

## Discussion

ABCs demonstrate a spectrum of clinical presentations, from the latent to the active form and finally the rare aggressive variant. The latter is sometimes extremely difficult to differentiate from telangiectatic osteosarcoma, as our case also clearly demonstrates. Referral to dedicated sarcoma centres, where clinical findings, radiology, cytology and pathology are interpreted by a multidisciplinary team offer clear advantages as far as diagnosis and therapy are concerned.

The biological aggressiveness of the lesion should be taken into account when choosing therapy, the more indolent forms can be treated with minimally invasive approaches or even observed, because there is a potential of spontaneous regression. As intralesional excisions have been associated with higher recurrence rate than wide excision, most authors have relied on a more radical resection and subsequent reconstruction of the skeletal defect to treat aggressive ABCs.

The presented case suggests that the non-invasive treatment with polidocanol is equally effective in aggressive ABCs, is not accompanied by side effects and seems to compare favourably to surgery. Indeed, morbidity associated to radical surgery is not negligible, time to recovery is often prolonged, and failures of the reconstruction have been reported [5]. Other methods also have drawbacks: selective arterial embolization is technically demanding and not applicable to all cases as the lesions often lack defined feeding vessels and radiotherapy carries the risk for development of late sarcomas [10]. Polidocanol sclerotherapy is, according to our experience [13] and previously published data [11, 12], a simple and safe treatment that can be performed on an out-patient basis, with no serious side effects and with very high efficacy.

## Conclusions

We suggest that polidocanol sclerotherapy be considered a treatment option for all ABCs, including the aggressive lesions. This is an important finding because it shows the applicability of a simple non-operative method for the treatment of a disease, which previously entailed open surgery with a considerable risk for side effects.

## Consent

Written informed consent was obtained from the patient for the publication of this case report and accompanying images. A copy of the written consent is available for review by the Editor-in-Chief of this journal.

## Abbreviations

ABC: Aneurysmal bone cyst
MRI: Magnetic resonance imaging
ROM: Range-of-motion.
